# Dizziness with a vestibular window on agency

**DOI:** 10.1007/s10072-021-05260-9

**Published:** 2021-04-21

**Authors:** Bauke M. de Jong

**Affiliations:** Department of Neurology, University Medical Center Groningen, University Groningen, P. O. Box 30 001, HpC-AB51 Hanzeplein 1, 9700 RB Groningen, The Netherlands

Dear Editor,

Vertigo includes the illusionary sensation of movement of either one’s body or surroundings. Due to vestibular neuritis, the author of this letter additionally experienced the remarkable perception of self-motion virtually inflicted by an external cause. This illusion gave rise to address brain functions implicated in the perception of self-control of one’s motor actions, i.e. the sense of agency, serving insight in the complaint worded by patients as ‘being pushed over’ [1].

Symptoms unfolded as follows. Half an hour preceding visual field motion, I was riding a bicycle when a short feeling of imbalance occurred of which I was convinced that it was due to a loose front wheel. No defect was seen. Subsequent vertigo with severe visual field motion lasted for about 3 days, which could only be slightly suppressed by fixating eyes to the left, reducing the ongoing nystagmus to the right. After improvement, bike riding still required enhanced attention. Head movements provoked a sense of imbalance, again attributed to a bike defect and not perceived as a result of myself deviating from the axial center of gravity. The cause of imbalance was projected to an external source. Misleading the neuronal mechanism of predicting the sensory consequences of movement may explain this phenomenon.

In cerebral motor control, cerebellar computations enable predictions on the sensory consequences of movement [2]. As motor output information is sent in parallel to sensory brain regions (‘efference copy’), predicted and actual sensory signals can be matched. If they match, afferent information can be cancelled: motor correction is not needed. Such feedforward processing strengthens sensorimotor integration which is strongly anchored in parietal circuitry. The forward model also explains why one cannot tickle oneself: the predicted sensation cancels the afferent tickle response to self-generated stimulation [3]. Closed-loop interconnection between the cerebellum and parietal cortex anatomically underscores this cerebellar contribution to sensorimotor processing. Vestibular input to the parietal cortex adds gravitational information serving spatial aspects of upright position and self-motion [4]. Mediated by cerebellar interactions, at both brainstem and cortical levels, vestibular information facilitates spatial predictions on self-motion [4].

Tuning predicted (feedforward) and actual (feedback) sensory information appears to be a key function associated with the perception of being in control of one’s motor actions, i.e. the sense of agency [5, 6]. Loss of such feeling, which may even turn into the illusion of alien action control, has been argued to result from misaligned actual and predicted action consequences [5, 7].

Upright standing and locomotion includes continuous axial adjustments made without thought. Impending loss of balance exerts an increased challenge on tuning actual and predicted vestibular and proprioceptive information. Now, a sense of imbalance arises, with corrections perceived as self-controlled adjustments to the center of gravity. In this, unity is still maintained between the perception that oneself is losing balance, i.e. sense of ownership, and sense of agency concerning the correcting movements [8, 9]. Sudden false vestibular information, dissociated from proprioceptive cues, gives rise to incomplete gravitation-based predictions with deteriorated spatial aspects of motor planning. Blurred predictions and the overruling dominance of actual stimuli thus cause an impaired perception of oneself being in control of the corrective balance movements. Failed prediction may actually induce the illusion that one’s movements are externally caused. This may explain ‘the unstable bike’ perception. Similarly, the model provides insight into vestibular attacks described by patients as if they are pushed over by an external agent, attacks referred to as Tumarkin falls [1].

To conclude, sudden vestibular misinformation leaves no time for gravitational predictions of axial balance movements at the level of cortico-cerebellar circuitry, a failure which may underlay the lost sense of agency. This would explain the vivid illusion of imbalance misattributed to an external cause, and serves better understanding of distinct dizziness-related complaints.

## References

[1] Ishiyama G, Ishiyama A, Baloh RW (2003). Drop attacks and vertigo secondary to a non-meniere otologic cause. Arch Neurol.

[2] Sokolov AA, Miall RC, Ivry RB (2017). The cerebellum: adaptive prediction for movement and cognition. Trends Cogn Sci.

[3] Blakemore SJ, Wolpert DM, Frith CD (1998). Central cancellation of self-produced tickle sensation. Nat Neurosci.

[4] Medendorp WP, Selen LJP (2017). Vestibular contributions to high-level sensorimotor functions. Neuropsychologia.

[5] Blakemore SJ, Wolpert DM, Frith CD (2002). Abnormalities in the awareness of action. Trends Cogn Sci.

[6] De Jong BM (2011). Neurology of widely embedded free will. Cortex.

[7] Voss M, Moore J, Hauser M, Gallinat J, Heinz A, Haggard P (2010). Altered awareness of action in schizophrenia: a specific deficit in predicting action consequences. Brain.

[8] Gallagher S (2000). Philosophical conceptions of the self: implications for cognitive science. Trends Cogn Sci.

[9] Pfeiffer C, Serino A, Blanke O (2014). The vestibular system: a spatial reference for bodily self-consciousness. Front Integr Neurosci.

